# Evolution of Moiré Profiles from van der Waals Superstructures of Boron Nitride Nanosheets

**DOI:** 10.1038/srep26084

**Published:** 2016-05-18

**Authors:** Yunlong Liao, Wei Cao, John W. Connell, Zhongfang Chen, Yi Lin

**Affiliations:** 1 National Institute of Aerospace, 100 Exploration Way, Hampton, VA, 23666, USA; 2 Department of Chemistry, Institute for Functional Nanomaterials, University of Puerto Rico, Rio Piedras Campus, San Juan, Puerto Rico, 00931, USA; 3 Applied Research Center, Old Dominion University, 12050 Jefferson Avenue, Newport News, VA 23606, USA; 4 Advanced Materials and Processing Branch, NASA Langley Research Center, Hampton, VA, 23681-2199, USA; 5 Department of Applied Science, The College of William and Mary, Williamsburg, VA, 23185, USA

## Abstract

Two-dimensional (2D) van der Waals (vdW) superstructures, or vdW solids, are formed by the precise restacking of 2D nanosheet lattices, which can lead to unique physical and electronic properties that are not available in the parent nanosheets. Moiré patterns formed by the crystalline mismatch between adjacent nanosheets are the most direct features for vdW superstructures under microscopic imaging. In this article, transmission electron microscopy (TEM) observation of hexagonal Moiré patterns with unusually large micrometer-sized lateral areas (up to ~1 μm^2^) and periodicities (up to ~50 nm) from restacking of liquid exfoliated hexagonal boron nitride nanosheets (BNNSs) is reported. This observation was attributed to the long range crystallinity and the contaminant-free surfaces of these chemically inert nanosheets. Parallel-line-like Moiré fringes with similarly large periodicities were also observed. The simulations and experiments unambiguously revealed that the hexagonal patterns and the parallel fringes originated from the same rotationally mismatched vdW stacking of BNNSs and can be inter-converted by simply tilting the TEM specimen following designated directions. This finding may pave the way for further structural decoding of other 2D vdW superstructure systems with more complex Moiré images.

“Nanosheets” is a general term referring to two-dimensional (2D) nanostructures typically with large lateral area but only one or a few atoms thick, such as graphene, hexagonal boron nitride (h-BN), and transition metal dichalcogenides^1^,^2^,^3^. The recent availability of various types of nanosheets makes it possible to prepare the so-called “van der Waals (vdW) superstructures” (or vdW solids, vdW junctions) by stacking up various nanosheets with the same (“homostructures” or “homojunctions”) or different (“heterostructures” or “heterojunctions”) chemical compositions via either direct growth or post-synthesis methods^4^,^5^,^6^,^7^,^8^,^9^,^10^,^11^. True vdW superstructures are ordered superlattices formed by nanosheets stacked at vdW distance over large overlapped lateral areas. The inter-nanosheet interactions due to their close proximity often result in unusual physical or electronic properties that are not available from individual nanosheets^12^,^13^,^14^,^15^,^16^,^17^.

To date, various microscopic and optical spectroscopic methods have been the primary tools to characterize vdW superstructures. However, verification of the formation of true vdW superstructures has been challenging. For example, optical spectroscopy, in combination with microscopy tools, has been successfully used to probe the electronic interactions between the vdW stacked nanosheet layers^18^,^19^,^20^,^21^, but it is intrinsically challenging to improve the resolution beyond sub-micrometer level due to the wavelength limitations of optical techniques. Alternatively, elemental mapping in electron microscopy with nanometer-level resolution can be used to characterize vdW heterostructures consisting of two or more types of nanosheets^4^,^5^,^6^,^7^,^8^,^9^,^10^,^11^,^12^,^13^,^14^,^15^,^16^,^17^. However, because the overlapped elemental maps are only indications of whether the different nanosheets are in the same path of the electron beam, such results can only serve as indirect proof for vdW superstructure formation.

A more definitive identification method of vdW superstructures is the visualization of Moiré patterns, which are ordered imaging projections of two or more ordered stacks of lattice sets (i.e., “superlattices”)^13^,^14^,^15^,^16^,^22^. The Moiré patterns often have a periodicity, sometimes referred to as “Moiré wavelength” or “Moiré lattice constant”, measured by the center distance of the neighboring unit features. Because of the ordered periodic characteristics, Moiré patterns can only form when the nanosheets are stacked at the vdW distance, and thus are direct proof of vdW superstructure presence.

Such Moiré patterns have been observed using various microscopic methods such as scanning tunneling microscopy (STM)^13^,^14^,^15^,^16^,^23^,^24^,^25^,^26^,^27^,^28^ and atomic force microscopy (AFM)^27^,^29^. For example, Moiré patterns have been commonly identified in STM as a result of the electronic interactions between the surface layers of nanosheets. These patterns are formed due to either structural mismatch between nanosheets of different chemical compositions (e.g., graphene–h-BN heterostructures)^8^,^22^,^25^,^26^ or rotational mismatch of identical nanosheet lattices (e.g., graphene-graphene rotational faults)^12^,^30^.

Moiré patterns are also commonly observed by transmission electron microscopy (TEM) and formed by the optical-like projection of transmitted electron beams owing to superimposed crystal lattices in the beam path^31^,^32^. By using high-resolution TEM (HR-TEM), typically with aberration-corrected equipment, Moiré patterns of the vdW homostructures (e.g., graphene^33^,^34^,^35^,^36^,^37^,^38^,^39^,^40^, h-BN^41^, MoS_2_^42^,^43^, MoSe_2_^44^,) and heterostructures (e.g., Bi_2_Se_3_/h-BN^45^) have been observed. In comparison with STM and AFM, which are shallow surface techniques, the comparatively less time-consuming TEM technique is rather useful to analyze objects through thicknesses up to tens of nanometers due to electron penetration.

A direct utilization of the Moiré pattern analyses is to derive the relative rotation angle of the stacked nanosheets (inversely related to Moiré periodicity; see the following text), which is one of the key structural characteristics of vdW superstructures that directly determines the electronic and spectroscopic properties. In a seminal work, Warner *et al.* deconvoluted the complex rotational faults from multi-layered stacking of graphene sheets via detailed analysis of the corresponding fast Fourier transform (FFT) images^38^. The relative rotational angles among the various layers of nanosheets were revealed by the hexagonally positioned spots in FFT, thus providing a detailed structural mapping of the superstructure. The concept has since been extensively applied to the vdW superstructure characterization, such as solving the relative rotation between the two monolayers in a bi-layer graphene (one of the simplest types of vdW superstructures)^37^,^38^ and grain boundaries for multi-stacked nanosheets with large lateral area^46^,^47^.

Liquid exfoliation methods using organic solvents or water are popular in the top-down preparation of various nanosheets from their parent layered crystals^48^. Such preparations typically involve the use of sonication, which often results in the size reduction and the loss of long range crystallinity of the resultant exfoliated nanosheets. Sonication can also partially decompose the solvent or other reagents used (such as surfactants), and the nanosheet surfaces are thus prone to contamination.

Intuitively, vdW superstructures from liquid-exfoliated nanosheets with large lateral stacked area should be rare. In fact, the majority of the prior reports on vdW superstructures using TEM characterizations have focused on Moiré patterns with lateral dimensions <100 nm and periodicities typically <5 nm^33^,^34^,^35^,^36^,^37^,^38^,^39^,^40^,^41^,^42^,^43^,^44^,^45^,^46^,^47^. Herein, TEM observations of Moiré features with unusually large lateral dimensions and periodicities from vdW homostructures formed by the restacking of liquid exfoliated BNNSs are reported. The Moiré features could be either hexagonal spots or parallel lines, but were found to be inter-convertible by tilting the projections of the nanosheets. The mechanistic origins and evolutions of such Moiré features were corroborated with simulations, as discussed in detail in the following text.

## Results and Discussion

### “Mesoscale” Hexagonal Moiré Patterns from Restacked BNNS Superstructures

Moiré patterns of restacked BNNSs were commonly observed in TEM investigations of specimens prepared from BNNS dispersions from direct liquid phase exfoliation (i.e., no surfactant) using solvents such as *N*,*N*′-dimethylformamide (DMF)^49^,^50^, water^51^, and tetrahydrofuran (THF). During solvent evaporation of the dispersions when preparing TEM specimens, restacked BNNSs were readily formed. Although not within the scope of the current work, a general observation was that the likelihood to observe vdW superstructures from an aged dispersion was typically higher.

**Figure 1:**
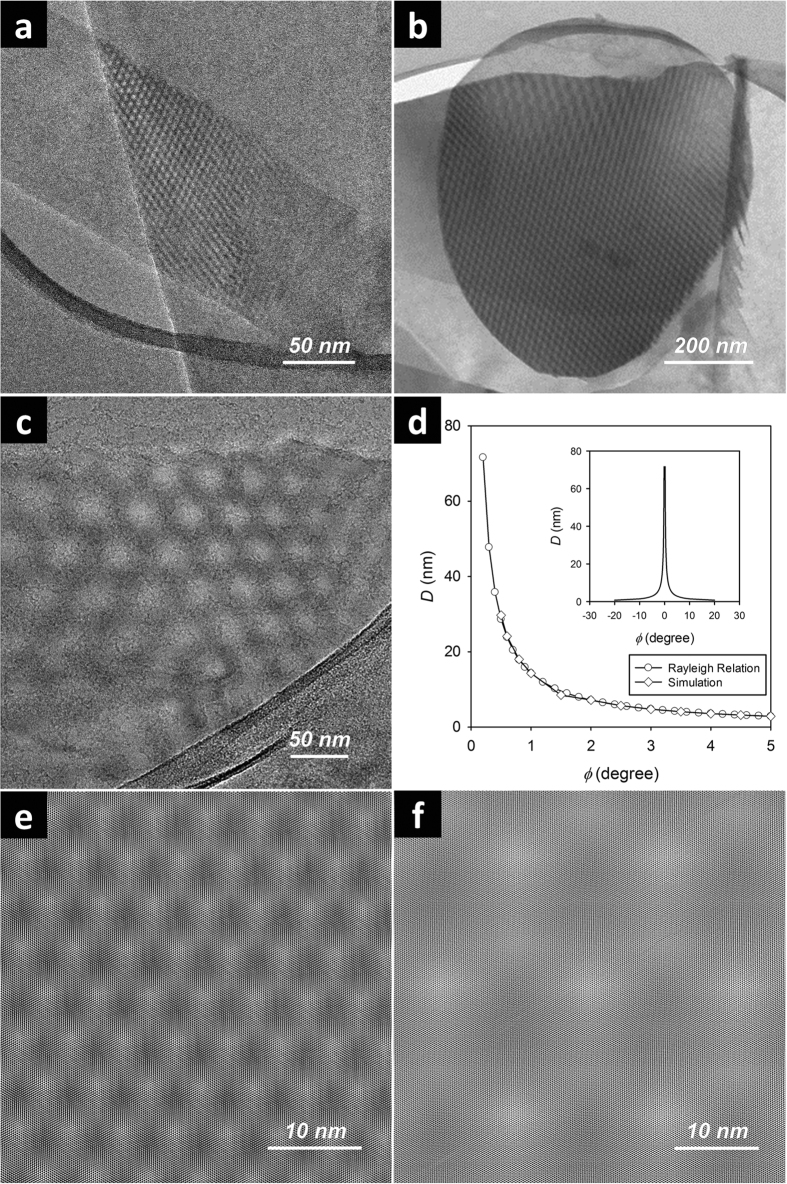
“Mesoscale” Moiré patterns of BNNS vdW superstructures: (**a–c**) low magnification TEM images of examples of Moiré patterns with various periodicities formed from large-area restacking of liquid exfoliated BNNSs; (**d**) dependence of Moiré periodicity (*D*) vs. the inter-nanosheet rotation angle (*ϕ*) at *ϕ* < 5° predicted by Eq. (1) (solid line) and simulation experiments (◊); the inset shows the entire periodic boundary (−30° < *ϕ* < 30°); (**e,f**) are simulated graphs of two restacked 10-layer BNNSs with rotation angles of 2.59° and 0.82°, corresponding to the calculated values from periodicities measured in (**a,b**), respectively.

Many observed patterns were hexagonal in nature, with varied Moiré periodicities (*D*_0_) ranging from 2–40 nm (similar in scale for “mesopores”) and ordered dimensions on the order of 100 nm–1 μm (similar in scale for “mesophysics”), as shown in Fig. 1. In Fig. 1a, while there was no pattern at the non-overlapped regions of the two BNNSs, a significant portion of the overlapped region exhibited ordered hexagonal patterns with dimension scales over 100 nm and *D*_0_ of 5.54 nm. The example in Fig. 1b is even more striking, as the ordered hexagonal pattern spanned over nearly 800 × 800 nm with *D*_0_ of 17.4 nm. The *D*_0_ value in the example in Fig. 1c was measured to be 44.5 nm, the largest periodicity observed in this work (and also perhaps reported in the literature). Note that it was not necessary to use high-resolution equipment to visualize the large scale periodicity patterns as they were readily discernible under low magnification. For example, the image in Fig. 1b was taken using a low-resolution instrument (Hitachi S-5200 field-emission scanning electron microscope under transmitted electron mode).

The mesoscale hexagonal patterns from the lattice mismatch in the vdW stacking of two BNNSs can be readily explained by the conventional rotational Moiré model. In this model, a Moiré pattern is formed by the rotational superimposition of two sets of lattices of identical or similar geometrical signatures (hexagonal for BNNS). The periodicity of the Moiré superstructure *D* with identical lattices can be described by:^15^,^31^,^33^,^52^,^53^


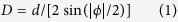


where *d* is the hexagonal lattice constant of a BNNS (0.250 nm) and *ϕ* is the rotation angle between the two restacked BNNSs. The boundary conditions of *ϕ* is set at −30° < *ϕ* < 30° due to the pseudo six-fold symmetry of the BNNS lattice because of the undetectable contrast distinction between B and N atoms with the equipment used in this work. As shown in Fig. 1d, the *D* value increases exponentially with the decrease of |*ϕ|*, especially when |*ϕ|* <2°. By using Eq. (1), the absolute rotational angles for the two restacked BNNSs in Fig. 1a–c were calculated to be 2.59°, 0.82°, and 0.32°, respectively. The relative simplicity of the observed Moiré patterns, despite the few-layered (typically 5–30 layers) nanosheet structures, could also be attributed to the dominating *AA*′-type stacking of the BN layers in each nanosheet. Different from the *AB*-type stacking commonly found in graphite and multi-layered graphene, all atoms of each honeycomb-like BN layer superimpose on those of the neighboring layers, with B “on top of” N or vice versa, as a result of the strong “lip-lip” (i.e., B-N) interactions^54^. In TEM, this renders a single set of hexagonal lattices as if it were from a monolayer. This characteristic makes the Moiré pattern observation and interpretation quite straightforward for the few-layered BNNS systems investigated in this work. With the use of HR-TEM, the “hidden” crystalline BN lattice structure within various hexagonal Moiré patterns was observed at higher magnifications. As shown in Figure S1 (Supporting Information), the restacked BNNSs exhibited large area hexagonal Moiré patterns with *D*_0_ of 5.54 nm (or a |*ϕ|* of 2.59°). At higher magnifications, the crystalline lattice became apparent. The atomic constant of h-BN was measured to be 0.25 nm, consistent with the expectations^54^.

As reported previously^33^,^34^,^35^,^36^,^37^,^38^,^39^, large rotation angles can be conveniently confirmed by the identification of hexagonally positioned spots from FFT conversion of the images. However, it was impractical to conduct similar FFT conversions of patterns originated from small rotation angles (1° or less), because the neighboring hexagonal FFT sets are in near-overlapped positions. Thus, simulation experiments were carried out (see the Supporting Information for the detailed method) to confirm the rotation angles calculated from Eq. (1). The simulated rotations were performed by using two 10-layered BNNSs starting from perfect *AA*′ stacking with layer-layer distance of 0.33 nm, the known vdW distance for h-BN. The distance of the two nanosheets was also set at 0.33 nm. The top nanosheet was rotated clockwise while keeping the bottom sheet immobilized. It was observed (see Movie S1 in Supporting Information) that the hexagonal pattern starts to occur from the nanosheet boundary at a large periodicity, which gradually decreases with the increase of rotation angle. The *D*-*ϕ* dependence perfectly matched that of the theoretical plot (Fig. 1d). By stopping the rotation angles at 2.59° and 0.82° in the simulation, hexagonal Moiré patterns with periodicities of 5.54 and 17.4 nm were re-created (Fig. 1e,f), matching the TEM observations shown in Fig. 1a,b.

Mechanistically, in order to form such mesoscale ordered patterns, several pre-requisites must be met. First, both nanosheets that contributed to the pattern must be highly crystalline and the interacting surfaces must be contaminant-free. Second, the stacking of two nanosheets must be highly ordered over a long range, including both uniform rotational lattice mismatch and uniform intersheet distance. The rotational lattice mismatch is required to form the hexagonal Moiré patterns, and the intersheet distance uniformity can be readily attributed to the vdW restacking. The folding back of a nanosheet onto itself to form an ordered superstructure is mechanistically and conceptually similar to the vdW restacking of two nanosheets, for which the previously described rules would also apply in order to form Moiré patterns.

The BNNS restacking process that led to small rotational faults was likely a result of an interplay between the thermodynamic preference of perfectly *AA*′ restacked nanosheets and the kinetic formation of random agglomerations in a liquid dispersion (either spontaneously or upon solvent evaporation). Although the kinetic nature determines that it is highly unlikely for two exfoliated nanosheets to restack in a perfect order, the strong B-N interactions that resulted in *AA*′ stacking might have made the small lattice mismatch much more prone to occur than the case of restacked graphene nanosheets (much less stacking preference), for which large inter-sheet rotational angles were commonly observed.

Although hexagonal Moiré pattern observation is not uncommon, what is really striking is that the liquid exfoliated BNNS allowed the formation of highly ordered Moiré patterns at such ultra-large lateral scale, suggesting that the nanosheet surfaces must be extremely “clean”. In comparison, ordered mesoscale Moiré patterns were seldom observed in few-layered graphene and MoS_2_ nanosheets similarly prepared from liquid exfoliation experiments under similar conditions, likely because the nanosheets were less crystalline and more prone to contaminations, in addition to their lack of strong lip-lip inter-sheet interactions. Preliminary experiments were also conducted by solvent exfoliating a mixture of h-BN and graphite. However, only the stand-alone few-layered BNNSs (identified by HR-TEM imaging showing a single set of hexagon lattice with constant of ~0.25 nm) exhibited ordered Moiré features.

### Parallel Line-Like Moiré Fringes and Their Origin

**Figure 2:**
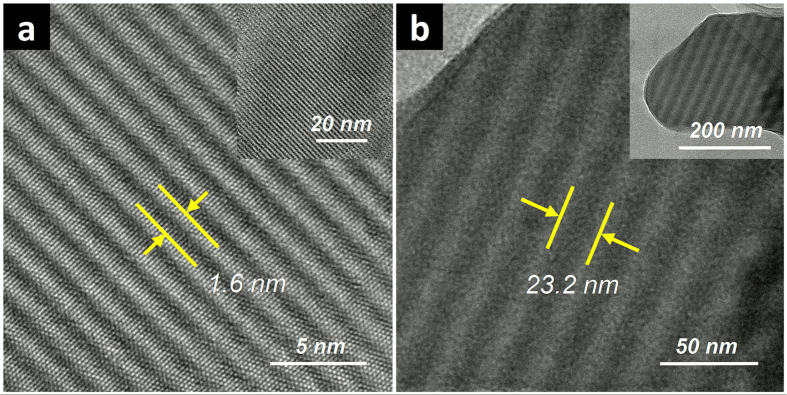
TEM images of parallel-line like Moiré fringes from BNNS vdW superstructures. Shown in the insets are the corresponding zoom-out images at lower magnifications.

Another common form of Moiré features from liquid exfoliated BNNS specimens found in this work was the parallel line-like fringes. Similar fringes have been observed before, but the exact origin was never convincingly addressed^55^,^56^,^57^. As shown in Fig. 2, the parallel fringes exhibited periodicities (i.e., distance between the centers of neighboring bright lines) ranging from a few to tens of nm, similar to those of the hexagonal patterns discussed previously. For example, the periodicities of the fringes in Fig. 2a,b were 1.6 and 23.2 nm, respectively. Again, the intrinsic hexagonal h-BN lattice (lattice constant 0.25 nm) was clearly visible at higher magnifications (Fig. 2a). In fact, in some cases where hexagonal Moiré patterns dominated (see Fig. 1a–c), certain areas of the mesoscale superstructures, most often close to their outer boundaries, exhibited parallel fringes that appeared as the natural extension of the hexagonal lattices. This observation seemed to indicate that the parallel fringes and the hexagonal patterns likely share the same origin, i.e., the formation of rotationally mismatched BNNS vdW superstructures due to restacking. It is thus hypothesized that the reason for the formation of these parallel fringes at the outer boundaries of hexagonal patterns might have been the physical deformation of certain parts of the stacked area, which resulted in distorted optical projections of the hexagonal patterns. The exclusively formed parallel fringes therefore most likely originated from highly uniform distortions of the optical projections.

**Figure 3:**
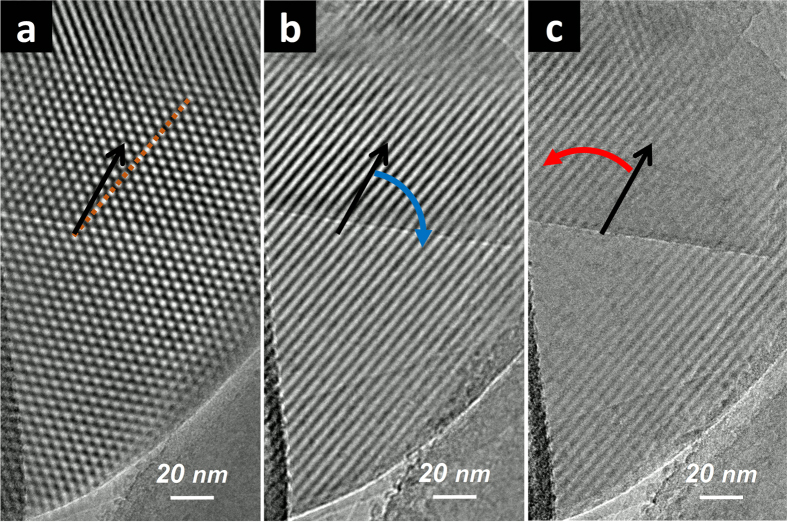
Evolution of mesoscale hexagonal Moiré patterns from BNNS vdW superstructures to parallel line fringes by tilting: (**a**) 0°; (**b**) + 4°; (**c**) −1°. The black-colored arrow indicates the tilting axis that slightly deviates from [110] (orange-colored dash line). The red and blue arrows indicate tilting toward *θ* > 0 and *θ* < 0 directions, respectively.

In order to prove this hypothesis, a hexagonal Moiré pattern was identified from vdW stacking of two BNNSs, as shown in Fig. 3a (the same one in Figure S1). The pattern has a hexagonal periodicity *D*_0_ of 5.54 nm, corresponding to a rotation angle |*ϕ|* of 2.59° between the two BNNS 2D lattices. An *in situ* experiment was then conducted by tilting the TEM specimen using a double-tilt holder (Model EM-31640 from JEOL). The tilting was in the *θ*_x_ axis (as indicated in the Figure S2 in Supporting Information) toward opposite directions (*θ* > 0 or *θ* < 0 following the right-hand rule). As shown in Fig. 3b, as a result of tilting toward the direction of *θ* > 0, a small increase of *θ* value (4°) resulted in the transformation of the hexagonal pattern into parallel fringes whose directions were close to the approximate axis of rotation ([110]). The periodicity of the parallel fringes (*D*_l_) was measured to be 4.61 nm, a value very close to 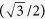
*D*_0_. Tilting the specimen toward the direction of *θ* < 0 (Fig. 3c) also resulted in parallel fringes with exactly the same direction and periodicity; however the pattern contrast was somewhat diminished possibly due to the object being tilted away from the available focusing depth.

The above experimental results unambiguously demonstrated that the parallel fringes and the hexagonal patterns are indeed inter-related and from the same origin: while the hexagonal patterns are from vdW superstructures with rotational mismatches that are vertical to the detector view, the occurrence of parallel fringes is the optical projection of the same superstructure but with a slightly tilted 2D plane.

### Simulated Evolution of Moiré Superstructural Profiles

**Figure 4:**
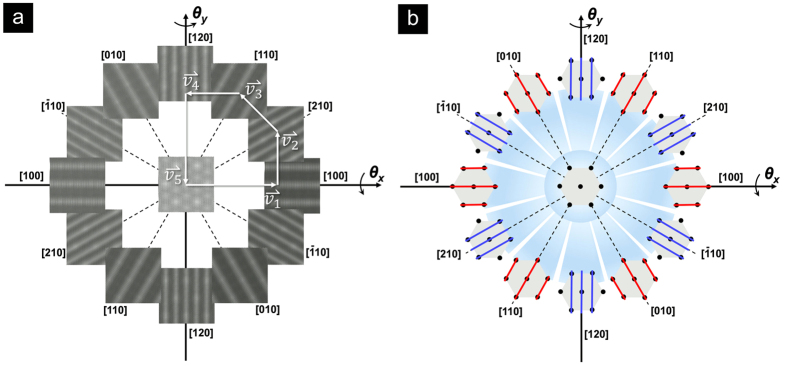
Complete evolution profile in a 2D angular coordinate system of Moiré features of BNNS vdW superstructures: (**a**) simulation results of various fringe lines obtained by tilting a hexagonal lattice (*D*_0_ = 5.54 nm) against the various axes shown (tilting directions follow the right-hand rule); (**b**) schematic illustration of fringe lines corresponding to the original hexagonal lattice.

The observation of Moiré superstructure evolution was further corroborated via comprehensive tilting simulations. As shown in the center image in Fig. 4a, a hexagonal Moiré pattern was first constructed by rotating a 10-layer *AA*′-stacked BNNS on top of another identical nanosheet with vdW distance of 0.33 nm and an offset of 2.59° from the perfect *AA*′ stacking position. As expected, the pattern periodicity *D*_0_ was measured to be 5.54 nm, consistent with the prediction from Eq (1). The entirety of the 20-layer vdW superstructure with the slight rotation mismatch was then tilted along [100], [210], [110], [120], [010], and 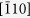 directions, respectively, following the right hand rule toward both *θ* > 0 and *θ* < 0 directions. In a 2D angular coordinate system (*θ*_x_,*θ*_*y*_), these directions can be described as (*α*,0), (*α*,*α*/2), (*α*/2,*α*), (0,*α*), (−*α*/2,*α*), (−*α*,*α*/2), respectively. As seen in the Fig. 4, tilting resulted in the removal of the optical degeneracy, and the gradual connection of the centers of the bright spots of the hexagonal patterns along the respective direction, forming parallel fringes with distinct periodicities that can be described by:


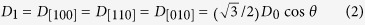



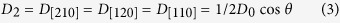


where *θ* is the true tilt angle defined by:


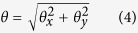


When |*θ|* < 8° (cos|*θ|* > 0.99 ≈ 1), the two periodicity values could be simplified into:


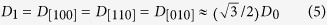



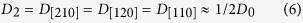


Therefore, parallel fringes from small angle tilting along [100], [110], and [010] directions share the same derived Moiré periodicity of *D*_1_, while tilting along the other three distinct directions also share the same periodicity of *D*_2_. The simplified schematics of this evolution profile is shown in Fig. 4b.

Tilting the hexagonal pattern along the axes between the above discussed distinct directions resulted in various pattern shapes that are intermediates of hexagons and parallel fringes from rotation along nearby distinct axes. Detailed information is given in the Supporting Information (Figure S3).

With the guidance of this principle, it is thus possible to tilt any given hexagonal pattern or parallel fringe with an appropriate tilting axis to transform it into the other Moiré pattern/fringe types with periodicity relationships defined by the previously described equations. The tilting axis is defined by the corresponding vector connecting the two pattern/fringe types in the 2D angular coordinate system. For example, as shown in Fig. 4a, the hexagonal pattern was tilted along the [100] direction to form a parallel fringe with an absolute tilting coordinate of (7.0, 0). This fringe was subsequently transformed into [210] [coordinate (7.0, 3.5)], [110] [coordinate (3.5, 7.0)], [120] [coordinate (0, 7.0)], and back to the original hexagonal pattern [coordinate (0,0)], respectively. The tilting axes were thus defined by the corresponding vectors 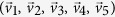 as also shown in the figure. A step-by-step transformation along similar vector paths was summarized into a movie file (Movie S2 in Supporting Information).

### Experimental Realization of Complete Moiré Profile Evolution

**Figure 5:**
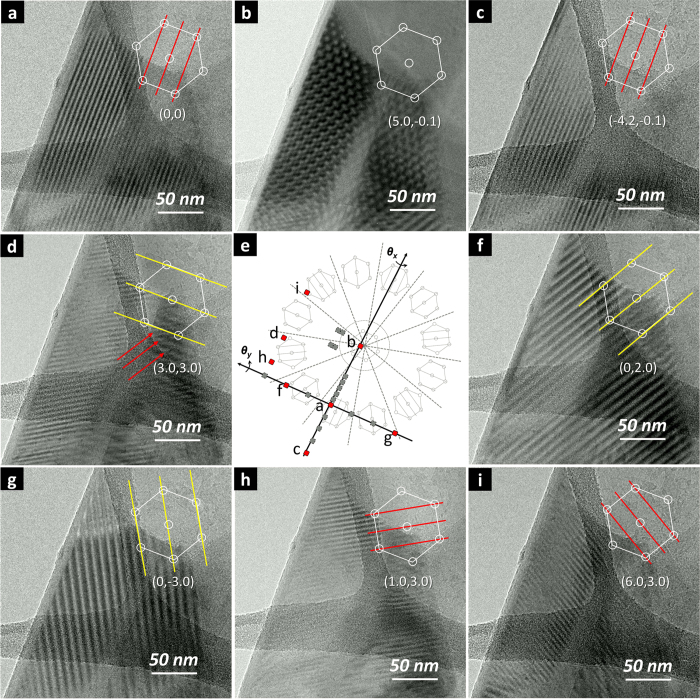
Comprehensive evolution of Moiré pattern and fringes of a BNNS vdW superstructure via free tilting experiments. The arrows in (**d**) point to the desired fringe directions. All tilting coordinates are superimposed onto the ideal schematics in (**e**). Images corresponding to the red-colored tilting coordinates are shown in this Figure, while those corresponding to the grey-colored ones are presented in Figure S4 in the Supporting Information.

The simulation results were achieved experimentally by completing the transformation of parallel fringes of all six distinct directions as well as their parent hexagonal pattern. The use of the same double-tilt specimen holder as that used for images shown in Fig. 3 was critical to allow the free tilting of the specimen with the orthogonal tilting coordinate (*θ*_x_,*θ*_y_) that defined the tilting vector *ν* in the 2D angular system. As shown in Fig. 5a, a distinct parallel fringe with periodicity of 5.41 nm from a self-stacked BNNS superstructure (nanosheet folded back onto itself) was initially identified. The parallel line exhibited a small angle (6.6°) vs. the *θ*_x_ axis. At this point, it was unknown whether this fringe was in the [100]/[110]/[010] category (where 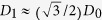) or in the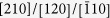 category (where 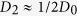).

According to the principle illustrated in Fig. 4a (similar to the case of Fig. 3), tilting the specimen along the parallel line direction would generate either the parent hexagonal Moiré pattern or parallel fringes of the same characteristics. This was exactly what was found. As shown in Fig. 5b,c, tilting the specimen along an axis near *θ*_x_ with vector coordinates of (5.0, −0.1) and (−4.2, −0.1) resulted in hexagonal Moiré pattern with a periodicity *D*_0_ of 10.6 nm (|*ϕ|* = 1.35°) and fringes with essentially unchanged characteristics, respectively. The *D*_0_ value suggested that the fringes in Fig. 5a,c were in the 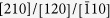 category 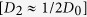. To represent the axis direction of these fringes, [210] was arbitrarily chosen, and the ideal tilting coordinate system (as illustrated in Fig. 4b) could be superimposed (in grey color) onto the actual coordinates (Fig. 5e).

Next, the specimen was tilted along the *θ*_y_ axis. As expected, tilting the specimen along the vector coordinates of (0, 2.0) and (0, −3.0) resulted in fringes with directions of [100] (Fig. 5f) and [110] (Fig. 5g), respectively, both with periodicity of 9.20 nm (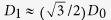). The TEM image from tilting along vector coordinates along (3.0, 3.0) exhibited fringes with directions of [010] (Fig. 5d, marked by red arrows) with periodicity of 9.20 nm. Further tilting the specimen along vector coordinates of (1.0, 3.0) and (6.0, 3.0) resulted in fringes with directions of 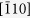 (Fig. 5h) and [120] (Fig. 5i), respectively. All tilting vector coordinates and the resultant patterns and fringes thus perfectly matched with the proposed 2D angular coordinate “map” (Fig. 5e). More exhaustive tilting experiments by gradually changing the *θ*_x_ and *θ*_y_ values (up to 6°) resulted in the observation of numerous patterns/fringes with intermediate characteristics (see Figure S4 in Supporting Information). As such, the various Moiré fringes and patterns observed could all be readily assigned to the same vdW BNNS superstructure slightly tilted along arbitrary axes. Similar tilting-induced fringe-pattern inter-conversions were achieved in multiple locations of the same specimen as well as other liquid exfoliated BNNS specimens.

These results suggested that, while the |*ϕ|* value for hexagonal patterns could be straightforwardly obtained via Eq. (1), those for the parallel fringes from vdW BNNS superstructures could fall in either the *D*_1_ or *D*_2_ category. A straightforward tilting experiment should help accurately determine the correct category and thus the correct |*ϕ|* value, considering that most nanosheets viewed in TEM are likely not placed perfectly horizontally but with a small tilting angle.

Finally, it should be noted that, the preliminary simulation data on the dependence of fringe-pattern inter-conversions on nanosheet layer numbers suggested that the parallel Moiré fringes exhibited higher “optical” contrast with thicker nanosheet layers, while the hexagonal patterns with no or little titling were less affected (Figure S5). More theoretical and experimental studies are needed to fully address this correlation.

In summary, vdW restacking of liquid exfoliated BNNSs resulted in mesoscale hexagonal Moiré patterns observed in TEM. These patterns had an ordered area as large as ~1 μm^2^ due to the long range crystalline order and defect/contaminant-free nature of liquid exfoliated BNNSs. Periodicities as large as ~45 nm were observed, which was due to the rotational mismatch in very small angles between two vdW restacked BNNSs. Another common type of Moiré feature observed under TEM on vdW BNNS restacked samples was parallel line-like fringes. It was demonstrated that these parallel fringes share the same origin as the hexagonal patterns, but only due to the removal of the degeneracy in optical projection via a small degree of tilting. Comprehensive tilting simulations and experiments demonstrated that the tilting directions strongly affected the fringe characteristics, which was straightforwardly illustrated using a 2D angular coordination system. These results suggest that the relative rotation angles of vdW superstructures forming parallel Moiré fringes in TEM can also be conveniently solved via a small degree of tilting. It is highly anticipated that the basic principle revealed in this article can be further extended into other vdW superstructure systems with more complex Moiré images^58^,^59^, and to identify and decode the exact stacking characteristics to correlate with their electronic and other physical properties.

## Methods

### Materials

h-BN powder (size -10P, Lot HZ010PA4.$06) was provided by UK Abrasives (Northbrook, IL). All solvents were used as received.

### Characterizations

The majority of TEM experiments were conducted on a JEOL 2100 field emission HR-TEM system at an accelerating voltage of 200 kV. Tilting experiments were conducted using a double-tilt specimen holder (JEOL Model EM-31640). The tilting directions were calibrated using an independent sample (see Figure S2). Some low-magnification TEM images were also acquired on a Hitachi S-5200 field-emission SEM system at an accelerating voltage of 30 kV.

### Exfoliated BNNS

In a typical procedure, the as-obtained h-BN powder (~20 mg) was sonicated in a selected solvent (DMF, THF, or water in this work; 20 mL) for 6–24 h using a bath sonicator (Branson 2510 Ultrasonic Cleaner, operating frequency at 40 kHz). The reaction flask was capped with a rubber stopper to avoid loss of volatile reagents. Upon completion of sonication, the resultant slurry was subjected to centrifugation at 3000 × g for 10 min to separate the supernatant dispersion from the residue. The supernatant was collected as the exfoliated BNNS. For TEM specimen preparation, a few drops of the as-prepared dispersion was placed onto a holey carbon–coated copper grid, followed by solvent evaporation under ambient conditions.

### Simulations

Material Studios (Accelrys) was used in all simulation experiments. Two 10-layer (unless otherwise specified) BNNSs, with inter-layer distance of 0.33 nm and *AA*′ stacking order, were used in the simulations. The vdW distance between the two sheets was set at 0.33 nm, which is also the inter-layer value for each nanosheet. The atomic radii for B and N atoms were both set at 0.06 nm, imitating the unresolved relative contrast of B/N atoms in TEM imaging. In the rotating experiments, the bottom BNNS was kept static, while the top nanosheet was rotated to a specific angle *ϕ* in a counterclockwise fashion. For the tilting experiments, the top nanosheet was first rotated counterclockwise to a desired angle as above, and then tilted in designated directions expressed through a 2D angular coordinate system (*θ*_x_,*θ*_y_) following the right-hand rule.

## Additional Information

**How to cite this article**: Liao, Y. *et al.* Evolution of Moiré Profiles from van der Waals Superstructures of Boron Nitride Nanosheets. *Sci. Rep.* **6**, 26084; doi: 10.1038/srep26084 (2016).

## Supplementary Material

Supplementary Information

Supplementary Movie S1

Supplementary Movie S2
