# Supplementary material for: Evolution of Moiré Profiles from van der Waals Superstructures of Boron Nitride Nanosheets
Source: Sci Rep. 2016 May 18;6:26084. doi: 10.1038/srep26084 (PMC4870578; doi:10.1038/srep26084)
Supplement: Supplementary Information [file srep26084-s1.pdf]

Supplementary Information

# Evolution of Moiré Profiles from van der Waals Superstructures of Boron Nitride Nanosheets

**Yunlong Liao,[1,2] Wei Cao,[3] John W. Connell[4], Zhongfang Chen,[2,*] and Yi Lin[1,5*]**

[1] National Institute of Aerospace, 100 Exploration Way, Hampton, VA, 23666, USA
[2] Department of Chemistry, Institute for Functional Nanomaterials, University of Puerto Rico, Rio Piedras Campus, San Juan, Puerto Rico, 00931, USA
[3] Applied Research Center, Old Dominion University, 12050 Jefferson Avenue, Newport News, VA 23606, USA
[4] Advanced Materials and Processing Branch, NASA Langley Research Center, Hampton, VA, 23681-2199, USA
[5] Department of Applied Science, The College of William and Mary, Williamsburg, VA, 23185, USA

Corresponding Authors: yi.lin@nianet.org; zhongfangchen@gmail.com

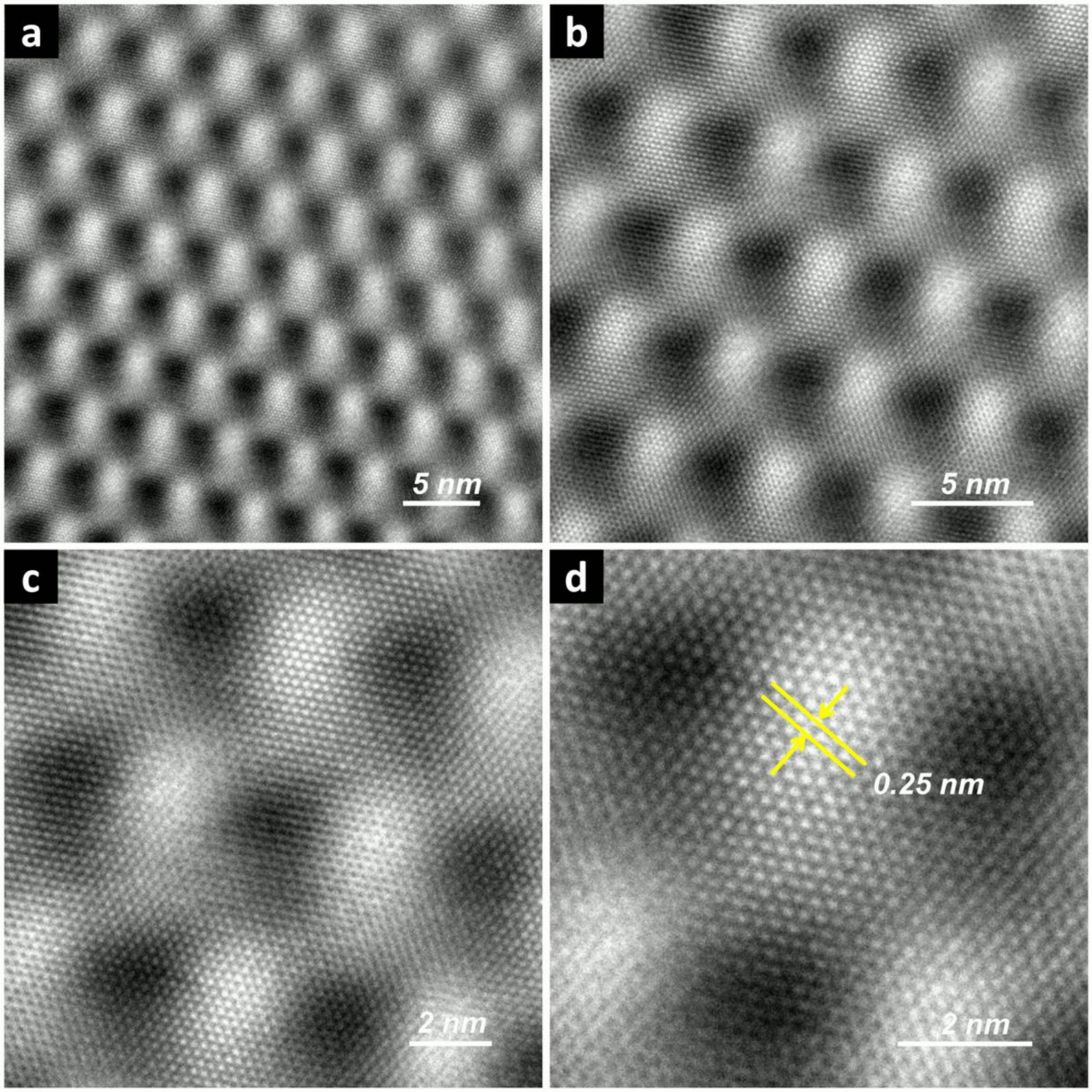


**Figure S1**. HR-TEM images with sequentially enlarged magnifications showing a hexagonal Moiré pattern of a restacked BNNS superstructure with a large Moiré periodicity (5.54 nm) and the underlying h-BN lattice with a constant of 0.25 nm.

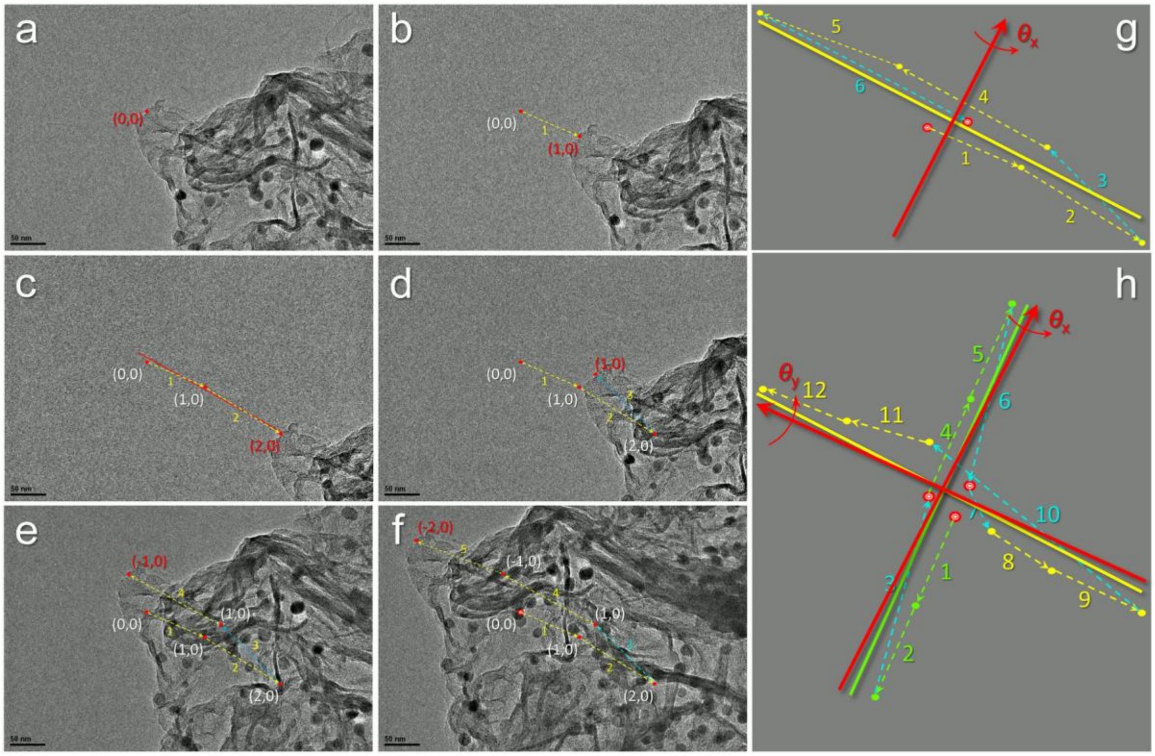


**Figure S2**. Identification of tilting axes of the double-tilt holder using an independent specimen (nickel oxide nanoparticle – decorated graphene). (a-f) are from an example of sequential tilting along $\theta_x$ axis in one (+) and then the opposite (-) directions. It was generally found that the image shift would generally follow a straight line with sequential tilting in the same direction (yellow color), but the immediate changing in tilting direction would result in a slight orientation shift [blue color; as shown in (d)]. The result was then extracted to (g), where the yellow line is the average direction of the image shift, suggesting that the $\theta_x$ axis (red) would be orthogonal to this direction. Another tilting sequence and the extrapolation of both $\theta_x$ and $\theta_y$ axes are shown in (h). The $\theta_x$ directions in (g) and (h) are of excellent match.

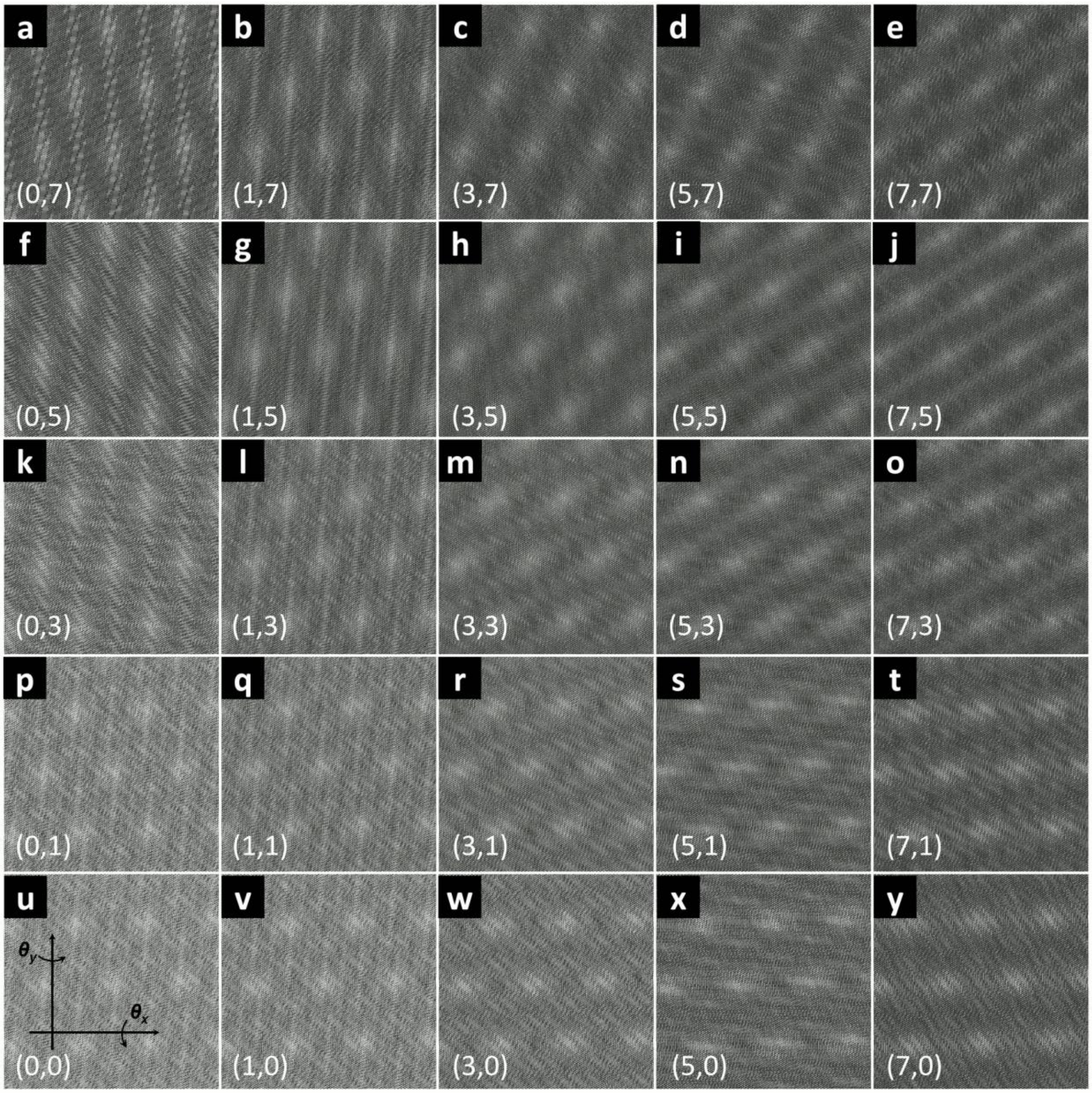


**Figure S3**. Simulated tilting results from various tilting degrees and orientations from a hexagonal Moire pattern ($D_0$ = 5.54 nm; the same system in Figure 4a) shown in the left bottom corner (u). The tilting coordinates are indicated in each image.

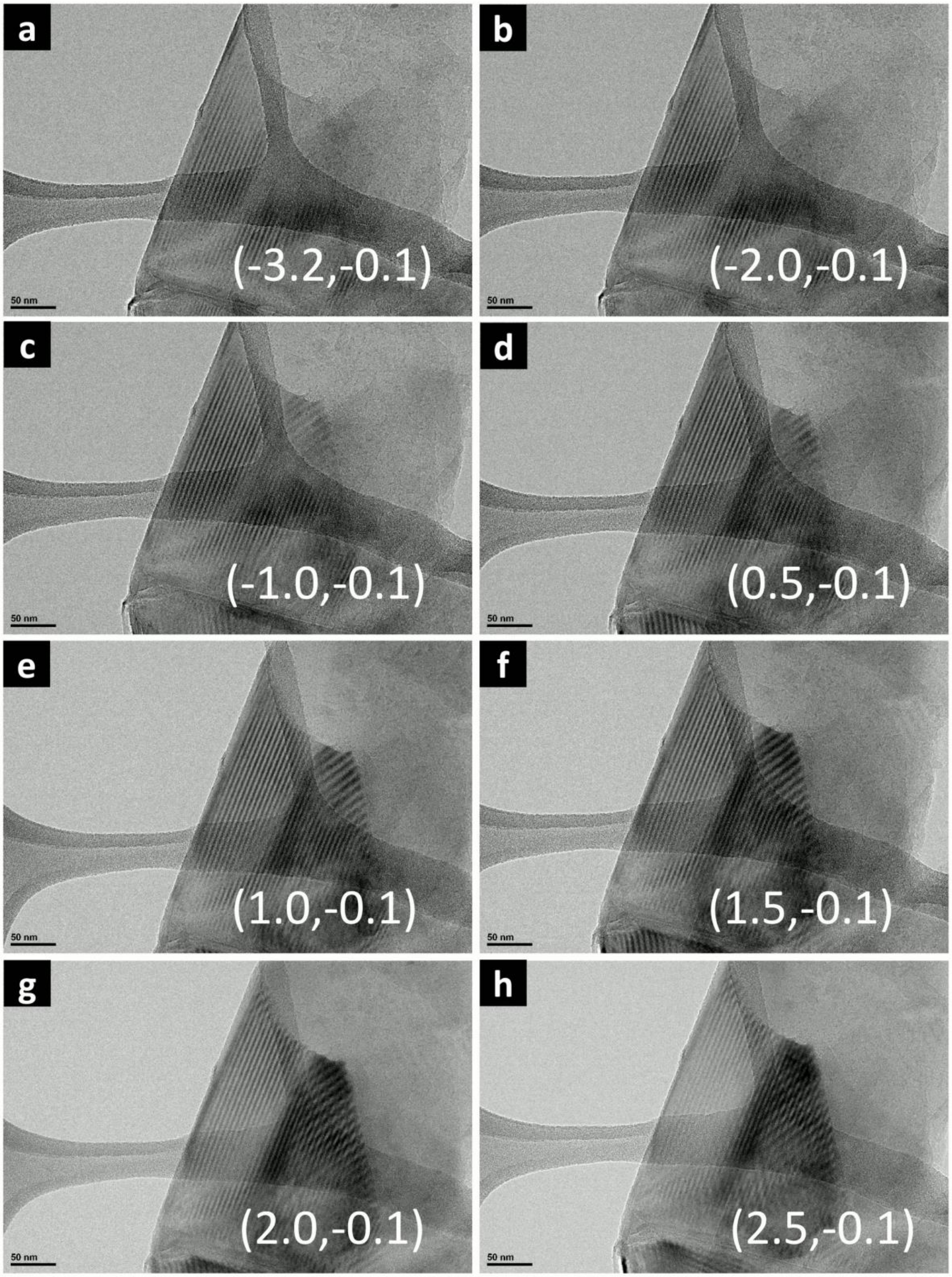


**Figure S4 – Part A**. TEM images from the Moire feature evolution experiment shown in Figure 5e. Tilting along $\theta_x$ axis (with a small $\theta_y$ value) resulted in almost exclusively the same characteristics as the original fringe feature in [210] direction.

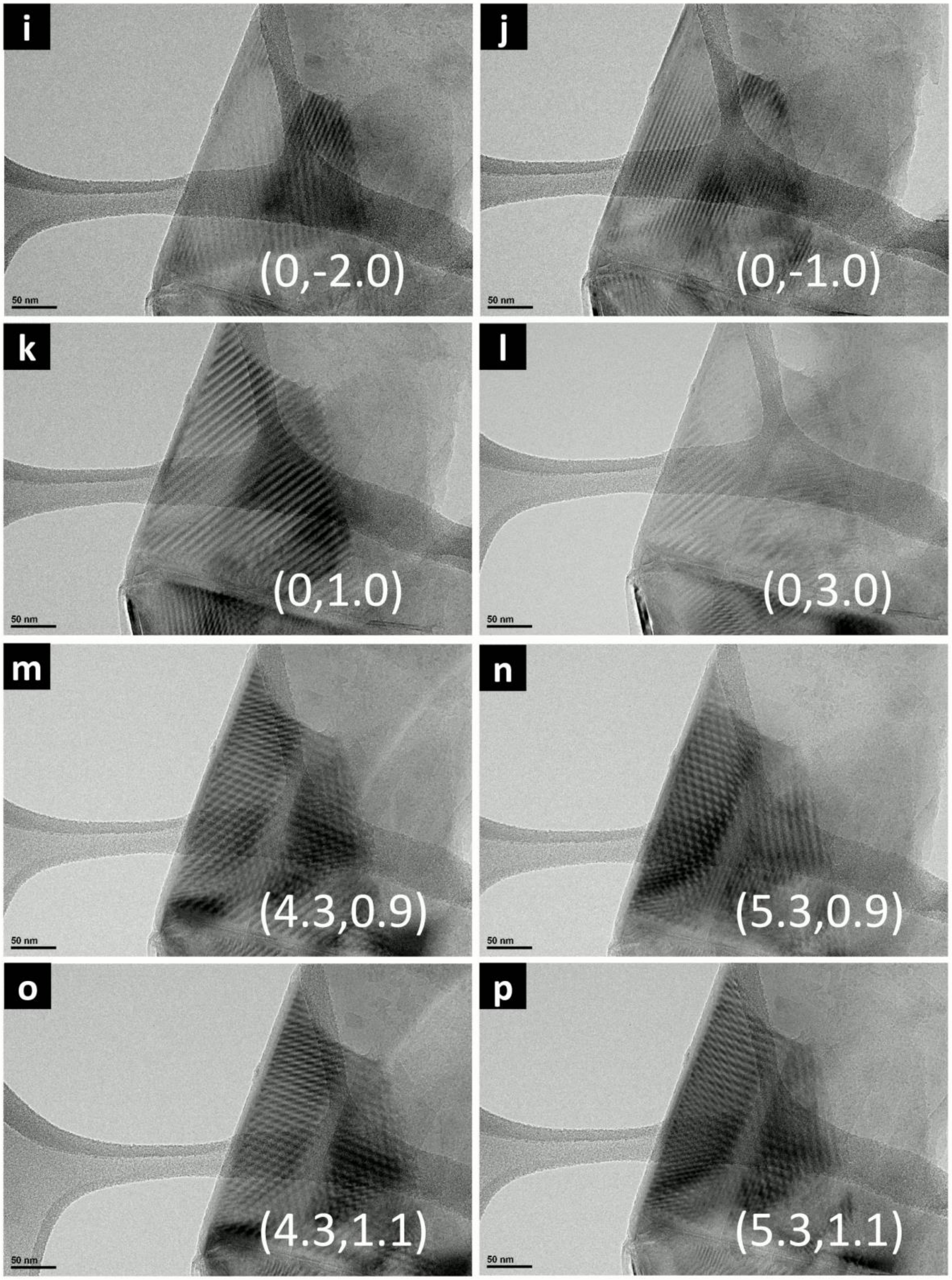


**Figure S4 – Part B**. TEM images from the Moire feature evolution experiment shown in Figure 5e. Tilting along other directions resulted in various fringe characteristics.

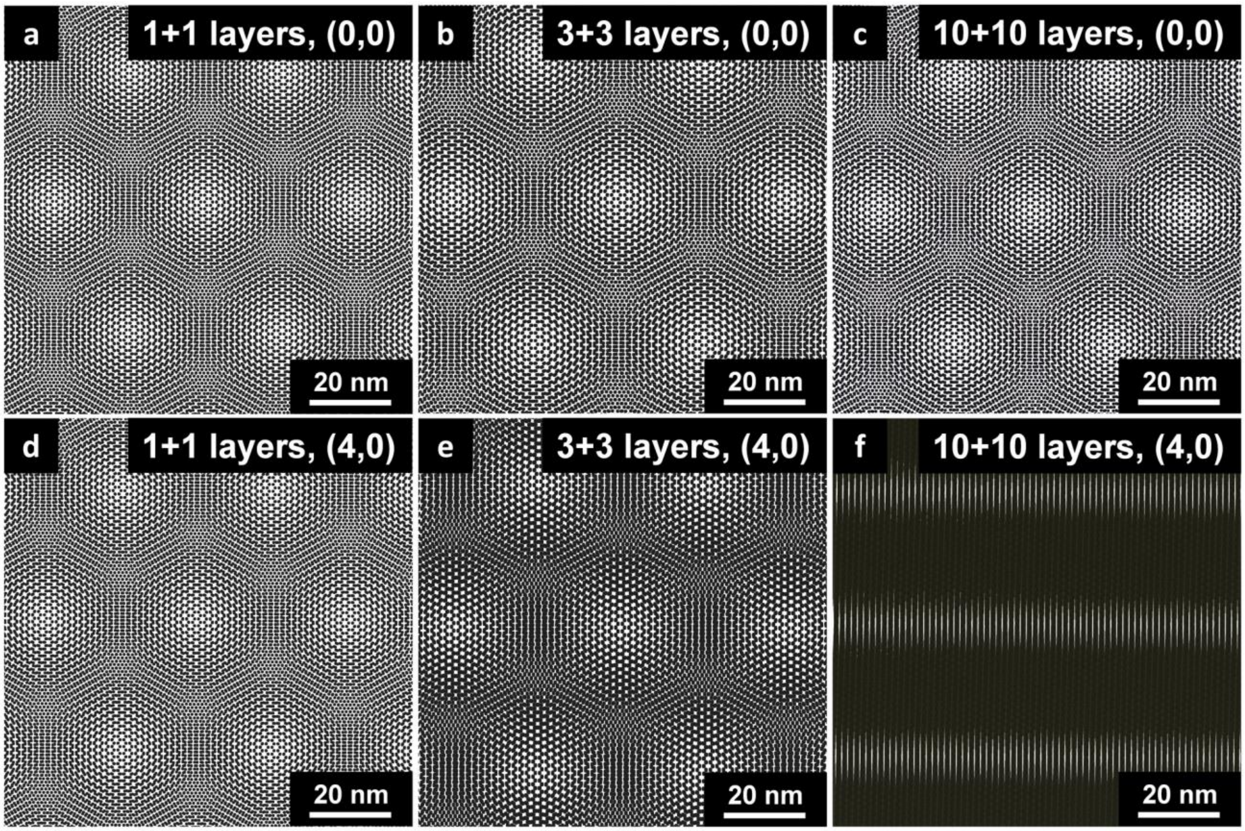


**Figure S5**. Simulations to show the effect of layer numbers on Moiré patterns and fringes of BNNS van der Waals superstructures using (a,d) (1+1)-layer, (b,e) (3+3)-layer, and (c,f) (10+10)-layer models. (a-c) are all hexagonal patterns each with a rotation angle $|\phi| = 0.4°$ ($D_0$ = 36.7 nm). (d-f) are the respective patterns/fringes that were resulted from a tilting coordinate of (4,0).

**Movie S1**. A simulation video on the evolution of the appearance of a hexagonal Moiré pattern of two stacked BNNS with the change of rotation angle (see the separate video file).

**Movie S2**. A simulation movie on the evolution of Moiré features, including both the hexagonal pattern and various parallel fringes, of two stacked BNNS with the change of tilting coordinates (see the separate video file).
